# Nodular Fasciitis of the Carpus Mimicking Metastasis in a Multi-cancer Survivor

**DOI:** 10.7759/cureus.101656

**Published:** 2026-01-16

**Authors:** Janse T Schermerhorn, Genevieve M Rambau

**Affiliations:** 1 Orthopaedic Surgery, Walter Reed National Military Medical Center, Bethesda, USA; 2 Orrthopaedics, Walter Reed National Military Medical Center, Bethesda, USA

**Keywords:** carpus, differential diagnosis, nodular fasciitis, soft tissue tumor, wrist mass

## Abstract

Nodular fasciitis is a benign reactive fibroproliferative lesion that is exceedingly rare within the wrist and hand, particularly when involving bone. Its rapid growth and clinical presentation can often mimic sarcomas or metastatic disease, creating a significant diagnostic challenge. We report the case of a 59-year-old male patient with a history of multiple malignancies who presented with a rapidly enlarging dorsal wrist mass. Advanced imaging revealed a large lesion eroding the carpus, raising significant concern for metastasis. However, histopathologic evaluation confirmed nodular fasciitis. The mass was excised en bloc without complex reconstruction. At the one-year follow-up, the patient remained asymptomatic with no clinical or radiographic evidence of recurrence and maintained excellent wrist function. This case highlights a rare anatomic location of nodular fasciitis and illustrates that it can mimic aggressive malignancy, particularly in cancer survivors. It further demonstrates that even with significant bony erosion, surgical excision without complex reconstruction can yield excellent functional outcomes.

## Introduction

Nodular fasciitis is a benign fibrous soft tissue neoplasm commonly caused by ubiquitin-specific protease 6 (USP6) rearrangements and often found in the forearm, upper arm, and back [1]. Generally, it is limited to soft tissues and is stratified based on the involved fascial layers. These lesions are characterized by rapid growth and are often initially confused with malignancy, specifically soft tissue sarcomas [1-5]. Physical exam and history may be helpful in diagnosis, but the initial differential is often broad. Advanced imaging, particularly MRI, is a critical part of the workup [6]. Ultimately, however, final diagnosis may be made by biopsy and subsequent histologic evaluation and is often supported by molecular analysis [4,5].

On rare occasions, nodular fasciitis is encountered in the wrist or hand, and even less commonly, it involves bone, with only scant reports in the literature [7,8]. Based on our literature review, we found a total of seven publications [9-14] describing histologically diagnosed nodular fasciitis of the hand (totaling nine cases) [15] and three in the wrist [16-18], two of which were inside the carpal tunnel, causing compressive neuropathy. We present a rare case of confirmed nodular fasciitis presenting with profound carpal bone erosion in a patient with a history of multiple malignancies. This report details the diagnostic challenge, the surgical treatment administered, and the patient's successful outcome without reconstruction. The patient was informed that deidentified data concerning the case would be submitted for publication, and he provided valid consent.

## Case presentation

A 59-year-old male patient presented to our institution with a dorsal wrist mass. The patient endorsed a rapidly enlarging mass over the preceding four months with an insidious onset. He sought care due to worsening pain that limited his activities of daily living and hobbies. The patient was initially worked up by his primary care physician, obtaining radiographs and an MRI. Notably, the patient had a significant history of malignancy, including testicular cancer successfully treated with orchiectomy, as well as medullary and papillary thyroid cancer, managed with total thyroidectomy and radioactive iodine. All thyroid cancer-related markers had been below clinically significant values since surgery, and the patient remained asymptomatic regarding his endocrine status.

**Figure 1:**
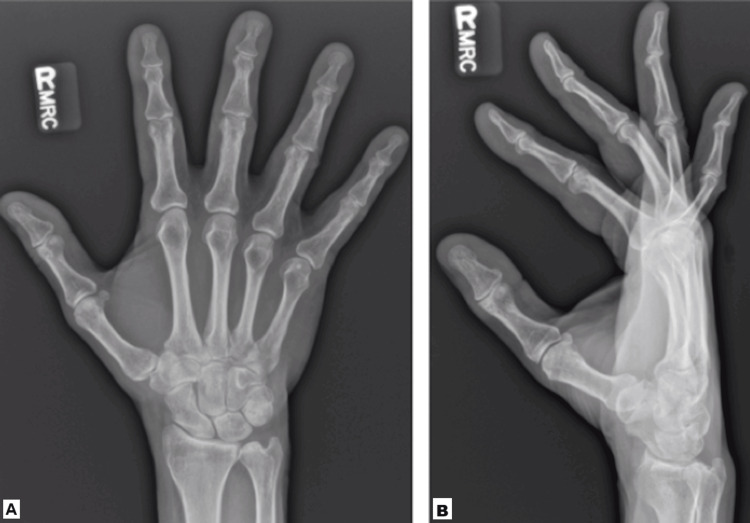
(A, B) Radiographs (AP, lateral) of the right wrist at presentation. No soft tissue shadow or osseous abnormalities are appreciated on plain film. AP: anteroposterior

The patient presented to the orthopedic hand surgeon with no systemic symptoms other than the continued enlargement of the wrist mass. Physical exam demonstrated a palpable, poorly circumscribed mass over the dorsum of the wrist. The overlying skin appeared unaffected. The patient had a full range of motion of the wrist and hands, but pain with passive and active wrist extension. No Tinel’s sign was elicited over the mass, and no pulsatile areas or bruits were appreciated. He had full strength and no neurovascular deficits. Radiographs did not demonstrate any soft tissue shadow of the wrist mass, nor were there other appreciated osseous abnormalities (Figure 1).

**Figure 2:**
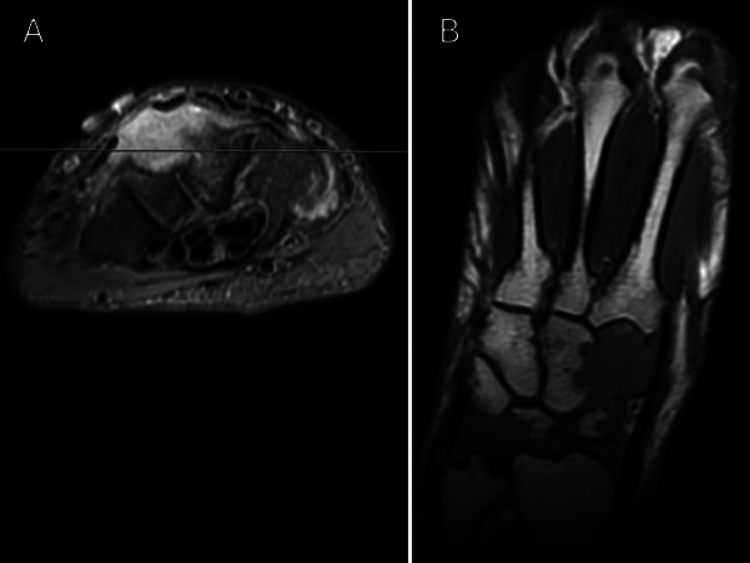
MRI of the right wrist with representative images of the dorsal wrist mass. (A) T2 hyperintense, heterogeneous mass deep to the ECRB and ECRL, appearing to erode into the capitate and trapezium. (B) T1 hypointense sequence re-demonstrating the mass eroding into the trapezium and capitate. ECRB: extensor carpi radialis brevis; ECRL: extensor carpi radialis longus

His MRI demonstrated a large (2.3 x 3.1 x 1.6 cm) poorly circumscribed T2 heterogeneously hyperintense and T1 hypointense mass that appeared to erode greater than 50% of the dorsal aspect of the trapezium, trapezoid, and capitate. The mass appeared deep and spared the extensor tendons (Figure 2).

**Figure 3:**
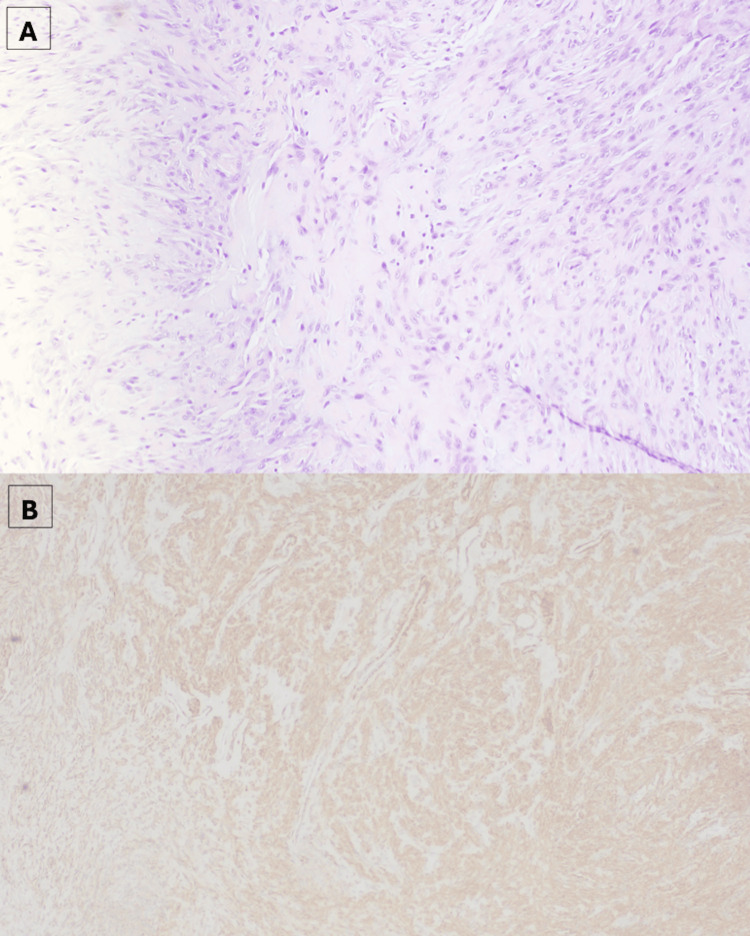
(A) Histopathologic image from biopsy of the patient's wrist mass, demonstrating spindle stellate cells on a myxoid background, as well as bland ovoid nuclei (hematoxylin-eosin staining, 10x magnification). (B) Immunohistochemical staining demonstrating diffuse positivity for smooth muscle actin (SMA), consistent with myofibroblastic differentiation (10x magnification).

Given the patient’s history of multiple malignancies and the destructive nature of the lesion, the differential diagnosis included metastatic disease or sarcoma. The patient underwent an incisional biopsy. Two 1 cm ellipses of tissue were removed and sent for fresh and frozen pathology evaluation. Pathology slides demonstrated spindle cells in a bed of collagenous stroma. The cells' immunohistochemical stains were negative for pancytokeratins (AE1/AE3, CK8/18), S100, SOX10, CD34, desmin, CD99, and BCL2, effectively ruling out metastatic carcinoma, neural tumors, leiomyosarcoma, solitary fibrous tumor, and synovial sarcoma. However, the spindle cells were positive with smooth muscle actin (SMA), leading to a pathologic diagnosis of nodular fasciitis (Figures 3A, 3B).

Given the locally destructive pathology and loss of normal wrist architecture secondary to bony erosion, the patient was indicated for mass removal. In the operating room, the mass was approached between the extensor carpi radialis brevis and longus. The mass was elevated off the dorsal carpus. It was noted that the mass had eroded away much of the dorsal capitate, trapezoid, and a portion of the trapezium. The scaphoid and metacarpals appeared unaffected. The mass was excised en bloc, measuring 4 x 2 x 2 cm. Following excision, there were no residual lesions or disruption of the remaining carpal architecture. The wrist was ranged, and the carpometacarpal joints and intercarpal ligaments remained stable. Therefore, the decision was made not to place any implants or perform fusion. Surgical pathology confirmed the previous diagnosis of nodular fasciitis.

**Figure 4:**
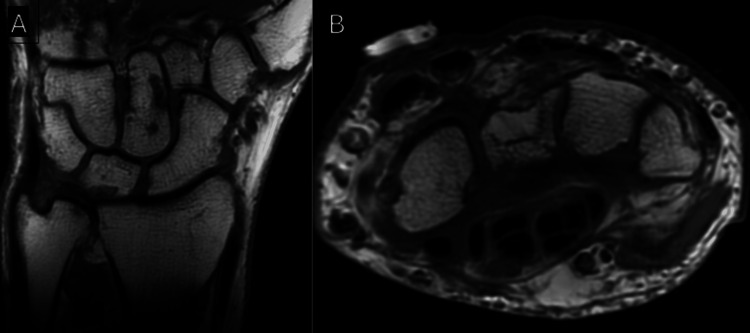
(A) Coronal and (B) axial MRI of the patient's carpus, 1 year post-resection, demonstrating no evidence of recurrence of nodular fasciitis.

Since surgery, the patient has had greater than one year of follow-up. He has done well clinically and radiographically, without signs of carpal instability. Repeat MRI scan at one year showed no further erosion of the carpal bones (Figure 4).

There has been no recurrence of the mass, and he has a normal hand/wrist exam with resolution of pain and return to all activities of daily living.

## Discussion

As a rapidly growing tumor, nodular fasciitis can cause significant concern for patients and physicians alike. Typically, this benign, self-limiting tumor does not cause significant structural issues and is often successfully treated with symptomatic care and observation [19]. However, here, we present a case that is unique for multiple reasons.

First, there are only a few reported cases of nodular fasciitis arising in the wrist, none of which had bony involvement as demonstrated in this case [16-18]. Bony involvement of nodular fasciitis in-and-of-itself is rare [14]. Park et al. described nodular fasciitis causing cortical disruption in the hand, specifically the proximal phalanx of the thumb [14]. They suggest that given its aggressive radiographic appearance, misdiagnosis can be common. This aligns well with literature describing nodular fasciitis mimicking metastatic disease or other malignant neoplasms [14,20]. Montreuil et al.’s case report described nodular fasciitis of the shoulder in this context and highlighted the importance of a multi-disciplinary approach to diagnosis and treatment, as well as future recognition of this pathology on the differential for rapidly expanding soft tissue tumors [20]. As a multiple-cancer survivor, our patient’s substantial bony erosions likely could have represented metastatic disease or a primary malignant soft tissue tumor. However, maintaining a broad differential and following fundamental orthopedic principles lead to a correct diagnosis of nodular fasciitis despite its rare location, subsequently allowing appropriate treatment with relatively conservative surgical excision.

Second, this case offers a valuable surgical teaching point regarding carpal stability in the context of benign neoplasms such as nodular fasciitis. Although we elected for surgical excision alone, other options would have included bone grafting or internal fixation, given the volume of bone loss. While massive carpal bone loss often suggests the need for reconstruction, this case demonstrates that the intrinsic stability of the distal carpal row, despite local tumor burden, can be remarkably forgiving. Provided the critical interosseous and extrinsic ligaments remain intact, tumor excision without ligamentous reconstruction remains a viable motion-sparing option.

## Conclusions

This case highlights a novel anatomic location of a relatively common benign neoplasm. Given only three reports of nodular fasciitis in the wrist, and none involving bone, a demonstration of a successful outcome despite surgically aggressive removal of the proximal carpal row is beneficial to those who may encounter similar situations in the future. Further, in the context of this patient’s multiple-cancer history and the unique location of the pathology, this case underscores the importance of maintaining a broad differential when confronted with an upper extremity mass. Continuing the discussion of new presentations of common pathologies serves as a critical reminder for surgeons to take the diligent, and often more time-consuming, route when working up their patients.
